# Supplementary material for: Pathway elucidation of bioactive rhamnosylated ginsenosides in Panax ginseng and their de novo high-level production by engineered Saccharomyces cerevisiae
Source: Commun Biol. 2022 Aug 2;5:775. doi: 10.1038/s42003-022-03740-y (PMC9345943; doi:10.1038/s42003-022-03740-y)
Supplement: Supplementary file 2 — Supplementary Information [file 42003_2022_3740_MOESM2_ESM.pdf]

Supplementary Information for:

Pathway elucidation of bioactive rhamnosylated ginsenosides in *Panax ginseng* and their *de novo* high-level production by engineered *Saccharomyces cerevisiae*

Chaojing Li[1, 2], Xing Yan[1], Zhenzhen Xu[1, 3], Yan Wang[1], Xiao Shen[1], Lei Zhang[4], Zhihua Zhou[1, *], Pingping Wang[1, *]

[1] CAS-Key Laboratory of Synthetic Biology, CAS Center for Excellence in Molecular Plant Sciences, Chinese Academy of Sciences, Shanghai, 200032, China

[2] University of Chinese Academy of Sciences, Beijing, 100049, China

[3] School of Life Sciences, Henan University, Kaifeng, 475001, China

[4] Logic Informatics Co., Ltd. Shanghai, 200031, China

These authors contributed equally: Chaojing Li, Xing Yan, Zhenzhen Xu

[*] Corresponding authors' email address: ppwang@cemps.ac.cn, zhouzhihua@cemps.ac.cn

Supplementary Methods

***Panax ginseng* transcriptome assembly.** Three different transcriptome assembly strategies were employed to construct comprehensive *Panax ginseng* transcriptome dataset. Method 1: 27 quality controlled transcriptomic datasets (Supplementary Table 10) were assembled using Trinity (version 2.5.1) for every single sample with default parameters. The resulted 27 transcript assembly dataset were shredded into set of sequences with a maximum of 2000 bp length and 300 bp overlap, Newbler (version 3.0) was used for co-assembly. The first non-redundant transcripts datasets was obtained using CD-HIT (version: 4.6.8). Method 2: 10 quality controlled transcriptomic datasets (Supplementary Table 11) were co-assembled using Trinity (version 2.5.1) to obtain the second transcripts dataset. Method 3: 27 quality controlled transcriptomic datasets (Supplementary Table 10) were aligned to reference genome (Ginseng_genome_assembly_v1.fasta.gz, Ref) using HISAT2 (version 2.1.0), StringTie2 (version 1.3.4) was used to reconstruct the third transcripts dataset. The annotated transcripts of the *Panax ginseng* genome project (Ginseng_transcripts_v1.fasta.gz) and another three assembled transcripts datasets were de-redunded to obtain the final set of transcripts dataset IPGI with CD-HIT (version 4.6.8).

The HPLC/mass spectrum (HPLC/ESIMS) analysis of Rg2 and Re. For MS analyses of the Rg2 and Re produced by yeast, an Agilent UHPLC-1290/MS QT0F6545A system equipped with Welch Boltimate C18 column (2.1*100 mm; 2.7 μm) was employed. The gradient elution consisted of ultrapure water (A) and

acetonitrile (B) used for washing and set as followed speed 0.45 mL/min, 0–7 min (17.5% B), 7–10 min (17.5%–24% B), 10–20 min (24% B), 20–21 min (24%–32.5% B), 21–25 min (32.5% B), 25–35 min (32.5%–65% B), 35–36 min (65%–95% B), 36–39 min (95% B), 39–40 min (95%–17.5% B), 40–43 min (17.5% B). The positive ionization mode was used for all MS analyses and scan range was between 100-1700 m/z. Other parameters of Dual AJS ESI were set as: drying gas temperature 300 ℃ at flow rate 6 L/min, and nebulizer pressure 30 psig, sheath gas 320 ℃ flow rate 11 L/min; the VCap 3500 V and Nozzle voltage 500 V. The TOF fragmentor was 140 V, skimmer 65 V and Oct RF 750 V. The collision energies were set 20 V and 40 V for acquiring ms/ms spectra. The [M+Na] + m/z values of Rg2 and Re are 807.4865, and 969.5393, respectively.

^{1}H NMR analysis of Rg2 and Re. The solvent was removed in vacuo and the resulted mixture was purified on column chromatography with macroporous resins, then the crude product was further purified with chromatography on silica gel (DCM/MeOH = 2/1) to obtain Rg2, and (EA/EtOH = 2/1) to obtain Re. NMR experiments were performed in pyridine-d_5 for Rg2 and Re on AVANCE III 400MHz Digital NMR Spectrometer (for ^{1}H NMR) (Bruker, Billerica, MA, USA) with reference to the solvent peaks.

Supplementary Figures

Supplementary Figure 1. Western blot analysis of PgURT94 expressed in *E. coli*. Marker, protein marker purchased from Yeasen Biotechnology(Shanghai， China); pET28a, crude enzyme solution of control strain containing empty pET28a vector; PgURT94Q3[1], crude enzyme solution of recombination strain pET28a- PgURT94Q3-BL21(DE3), PgURT94, crude enzyme solution of recombination strain pET28a-PgURT94-BL21(DE3).

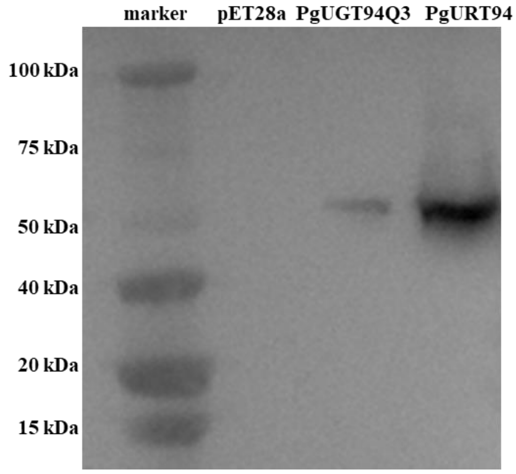

Supplementary Figure 2. HPLC/ESIMS analysis of ginsenoside Rg2 and Re produced by yeast strain Rg2-04 and Re-01, respectively. For Rg2 and Re authentic sample, the molecular weight of $[M+Na]^+$ is 807.4865 and 969.5393, respectively. (A) The mass spectrum of Rg2 produced by strain Rg2-04. (B) The mass spectrum of Rg2 authentic sample. (C) The mass spectrum of Re produced by strain Re-01. (D) The mass spectrum of Re authentic sample.

A.

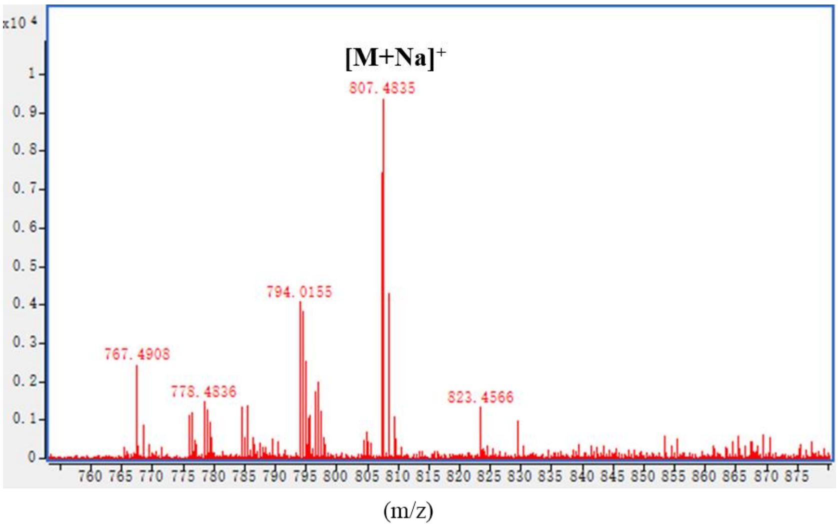

B.

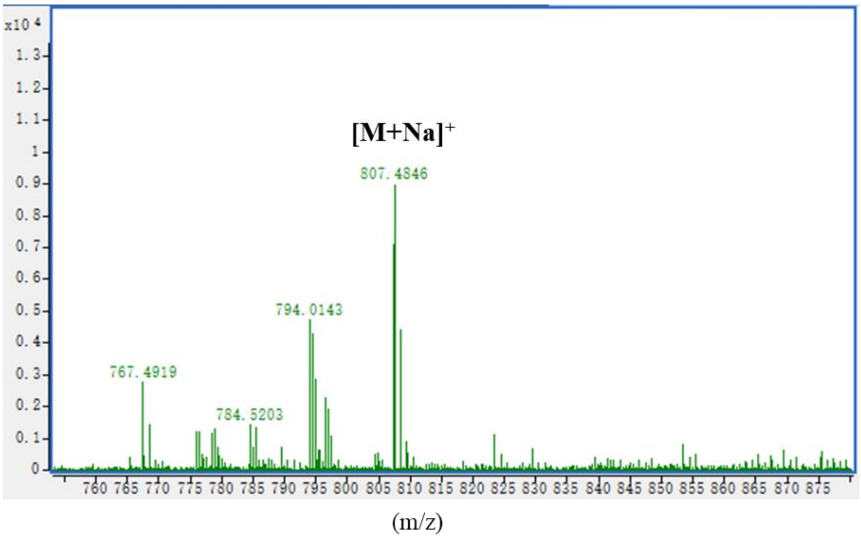

C.

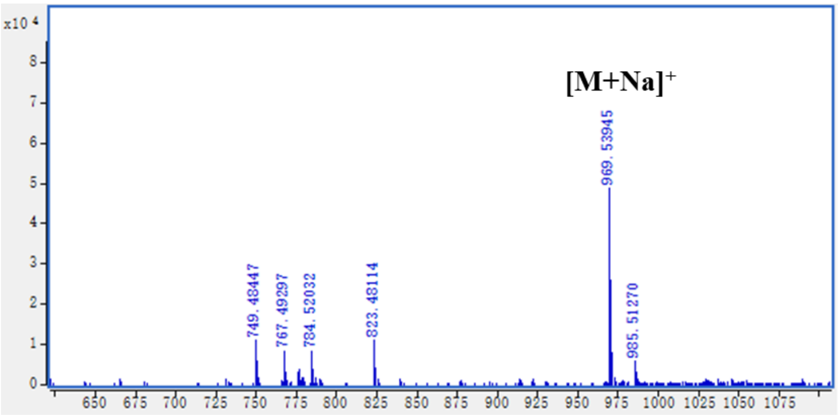
x10 4
8
7
6
5
4
3
2
1
0
650 675 700 725 750 775 800 825 850 875 900 925 950 975 1000 1025 1050 1075
[M+Na]+
969.53945
749.48447
767.49297
784.52032
823.48114
985.51270

(m/z)

D.

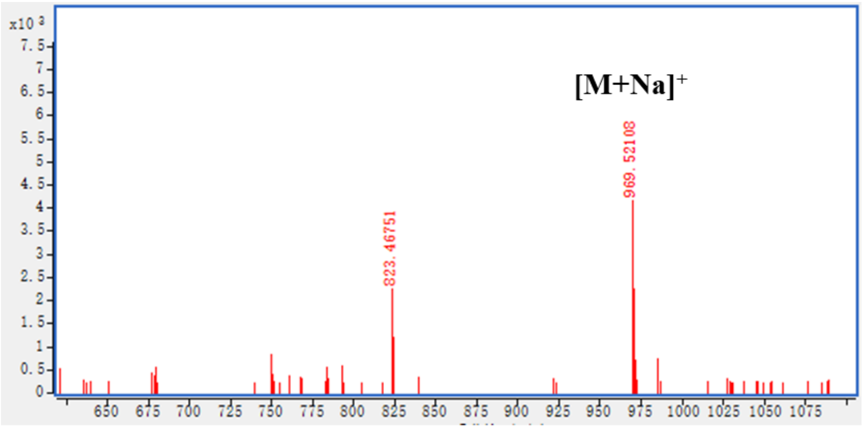
x10 3
7.5
7
6.5
6
5.5
5
4.5
4
3.5
3
2.5
2
1.5
1
0.5
0
650 675 700 725 750 775 800 825 850 875 900 925 950 975 1000 1025 1050 1075
[M+Na]+
969.52108
823.46751

(m/z)

Supplementary Figure 3. NMR analysis of ginsenoside Rg2 and Re produced by yeast strain Rg2-04 and Re-01, respectively. (A) The ^{1}H NMR of Rg2 produced by strain Rg2-04. (B) The ^{1}H NMR of Re produced by strain Re-01.

A.

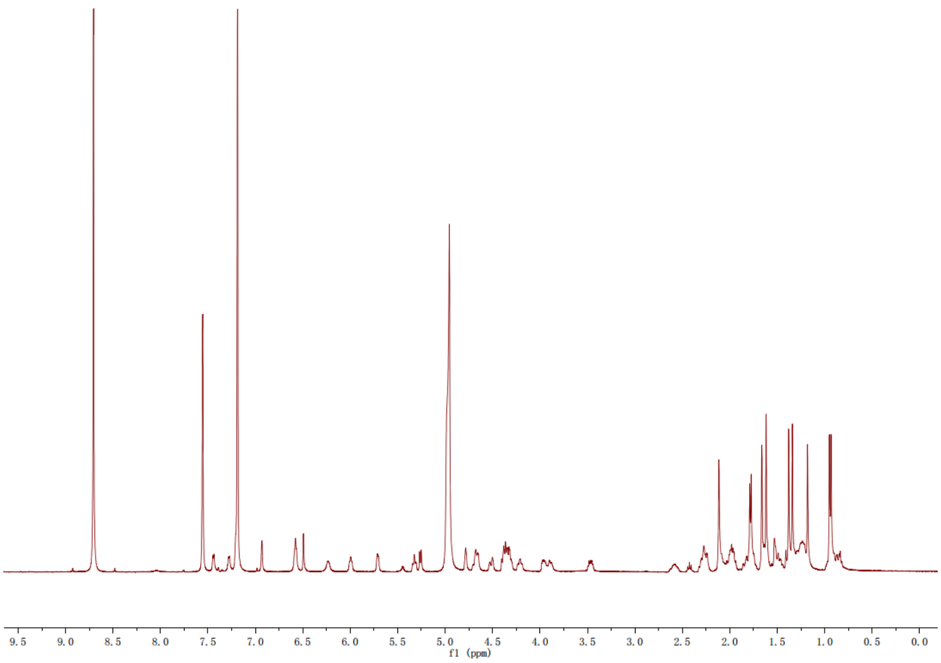

B.

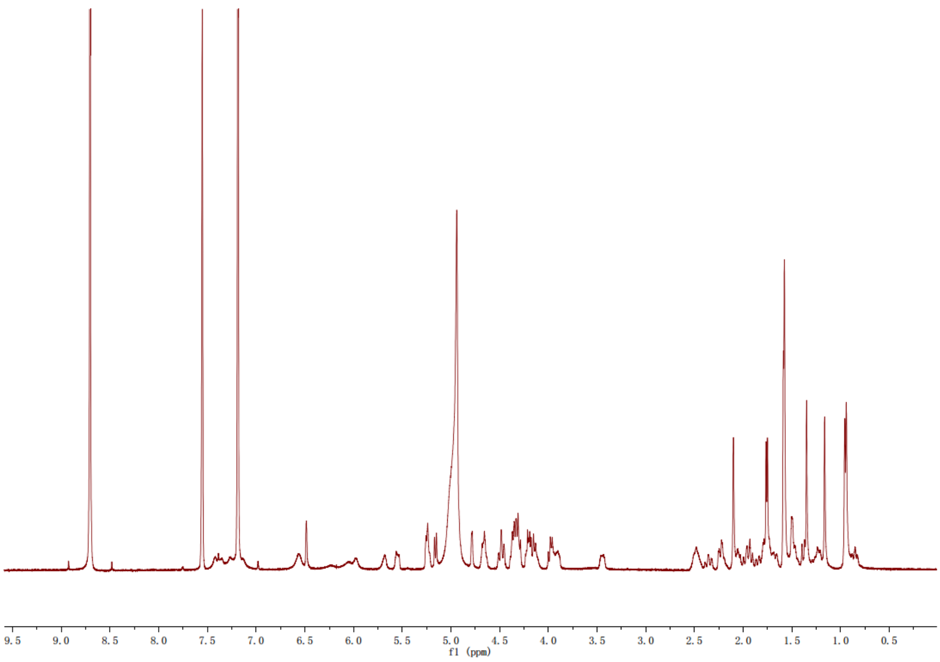

Supplementary Figure 4. Sugar donor specificity assay of PgURT94. a-b) Sugar donor specificity assay of PgURT94 using Rh1 as a sugar acceptor. TLC (a) and HPLC (b) analyses of the *in vitro* reaction products catalyzed by PgURT94 crude enzyme using Rh1 as sugar acceptor and UDP-glucose as a sugar donor. c-d) Sugar donor specificity assay of PgURT94 using Rg1 as a sugar acceptor. TLC (c) and HPLC (d) analyses of the *in vitro* reaction products catalyzed by PgURT94 crude enzyme using Rg1 as sugar acceptor and UDP-glucose as a sugar donor. Previous reported PgUGT94Q3 which could catalyze the formation of ginsenosides Rf and C20-O-Glc-Rf from Rh1 and Rg1 using UDP-glucose as a sugar donor, was used as a positive control. Crude enzymes of *E. coli* strain harboring pET28a empty vector were used as a negative control.

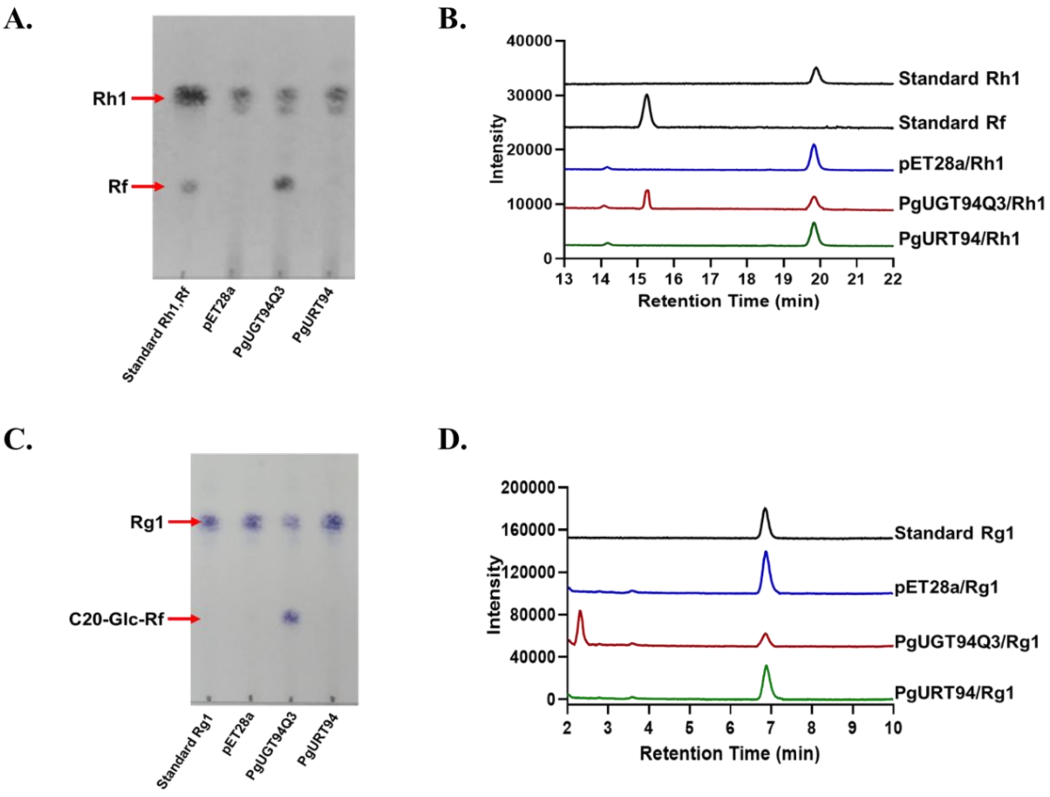

Supplementary Figure 5. Summary of reaction steps of ginsenoside biosynthetic pathway which required NADPH as co-factor. A. The formation of mevalonate (MVA) from 3-hydroxy-3-methylglutaryl-coA (HMG-CoA)[2]. B. The formation of 2, 3-oxidosuqalene from squalene[3]. C. Two P450s catalyzing the formation of PPT from dammarenediol-II[4, 5]. D. AtRHM2 catalyze the formation of UDP-Rhamnose from UDP-glucose[6]. E. The engineered VvRHM-NRS catalyze the formation of UDP-Rhamnose from UDP-glucose[7].

A.

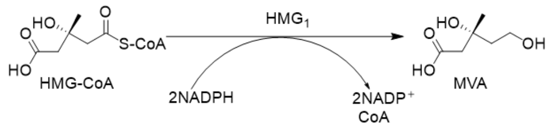

B.

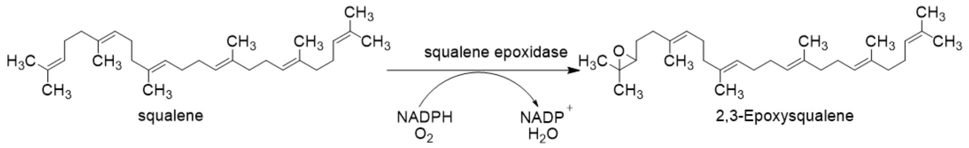

C.

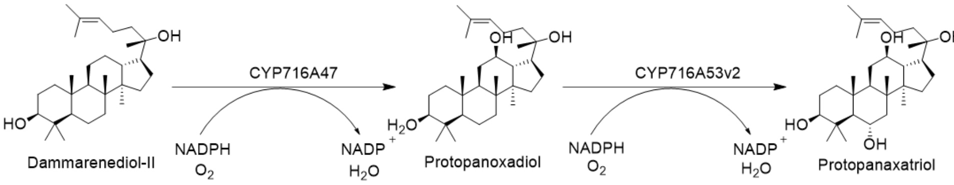

D.

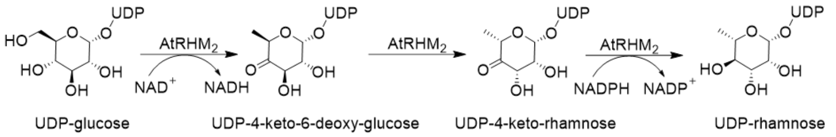

E.

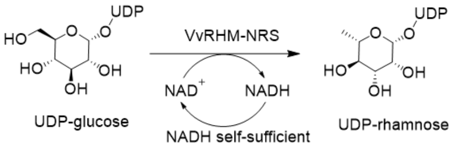

Supplementary Figure 6. Quantitative analysis of total triterpenoids production of Rg1-02 and Re-01. Data represent the mean of n = 3 biologically independent samples and error bars show standard deviation. PPD, protopanaxadiol, PPT, protopanaxatriol, DMG, 20*S*–O-*β*-(D-glucosyl)-dammarenediol-II.

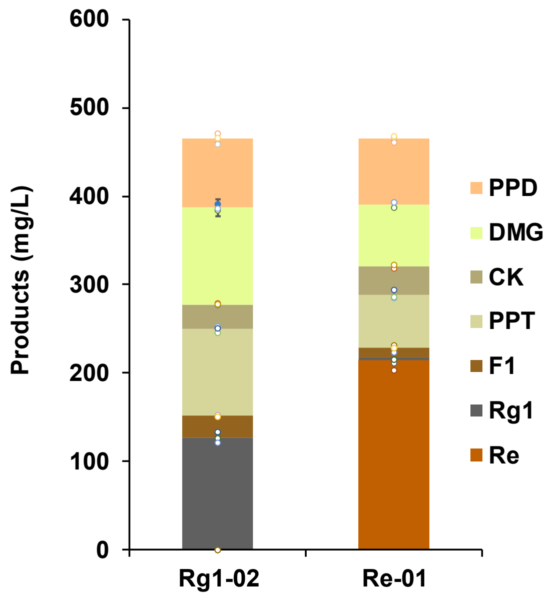

Supplementary Figure 7. Uncropped and unedited Western blotting image of Supplementary Figure 1. Lane 6, pre-stained protein marker; Lane 7-9, crude enzyme solution of control strain containing empty pET28a vector, PgURT94Q3 and PgURT94, respectively; Lane 1-5 & lane 10-12, other protein samples not related to this work, this data has not been and will not be published elsewhere.

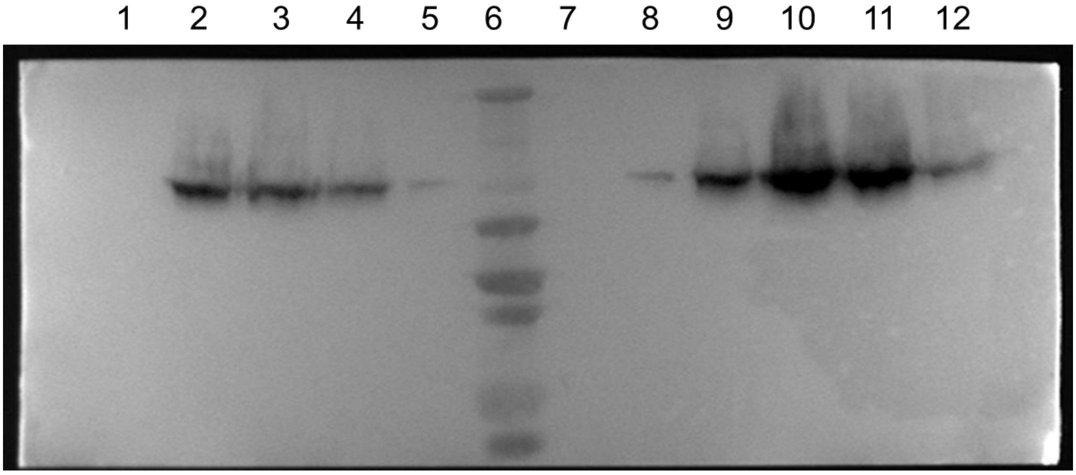

Supplementary Tables

Supplementary Table 1. Contents of Rg2 and Re in the *P. ginseng* root and leaf.

**Sources**	**Rg2 (mg/g, DCW)**	**Re (mg/g, DCW)**	**References**
Root hair	$6.3\times10^{-1} \pm 1.3\times10^{-2}$	$2.2 \pm 2.0\times10^{-2}$	[8]
Main root	-	$1.9\times10^{-1} \pm 1.2\times10^{-2}$	
Leaf	$1.8\times10^{-1} \pm 5.9\times10^{-3}$	$1.4 \pm 9.0\times10^{-3}$	

"-"：indicate not detected.

Supplementary Table 2. Primers and strains used for cloning and heterologous expression of URTs in this study.

Strains	Primers	Sequences (5' to 3')
pMD18-T-PgURT94-TOP10	E-01	TGCCTGCAGGTCGACGATATGGATACCAATGAAAAAACCA
	E-02	CGGGGATCCTCTAGAGATTTAGGGGCATCGCTTCCCCTGGCCTG
pET28a-PgURT94-BL21(DE3)	E-03	CTTTAAGAAGGAGATATACCATGGATACCAATGAAAAAACCA
	E-04	TCGAGTGCGGCCGCAAGCTTGGGGCATCGCTTCCCCTGGCCTG
pET28a-synPgURT94-BL21(DE3)/ pET28a-synPgURT94m1-BL21(DE3)	E-05	CTTTAAGAAGGAGATATACCATGGACACCAACGAGAAGACCAG
	E-06	TCGAGTGCGGCCGCAAGCTTTGGACACCTCTTACCTTGACCT

Supplementary Table 3. Yeast strains used in this study.

Strains	Genotype or characteristic	Sources
PPT-10	BY4742 (*delta DNA* :: *HXT7p-tHMG1-ADH1t, TEF2p-synPgCPR1-TDH2t, TPIIp-ERG1-ENO2t, GPM1p-ERG20-CYC1t, PGK1p-ERG9-FBA1t, TDH3p-synDDS-PGT1t, TEF1p-synPPDS-PGK1t; YPRCδ15* :: *ENO2p-ERG12-CPS1t, TEF2p-ERG13-IDP1t, TPIIp-ERG8-PRM5t, GPM1p-ERG19-HIS5t, PGK1p-IDI-PRM9t, TDH3p-ERG10-SPG5t, TEF1p-tHMG1-ADH1t; rDNA* :: *TDH3p-synPPDS-CPS1t; delta DNA* :: *UAS-TDHp-synPgCYP2m-FBA1t*)	[9]
Rg2-01	PPT-10(*delta DNA* :: *TEF2p-AtRHM2-TDH2t, TEF1p-PgUGT71A54-CYC1t, UAS-TDH3p-synPgURT94-FBA1t, Phe*)	This study
Rg2-02	PPT-10(*delta DNA* :: *TEF2p-VvRHM-NRS-TDH2t, TEF1p-PgUGT71A54-CYC1t, UAS-TDH3p-synPgURT94-FBA1t, Phe*)	This study
Rg2-03	PPT-10(*delta DNA* :: *TEF2p-VvRHM-NRS-TDH2t, TEF1p-PgUGT71A54-CYC1t, UAS-TDH3p-synPgURT94m1-FBA1t, Phe*)	This study
Rg2-04	Rg2-03(*rDNA*:: *UAS-TDH3p-sPgUGT71A54-ENO2t,nat*)	This study
Rg1-02	PPT-10 (*delta DNA* :: *TEF1p-PgUGT71A54-ENO2t, UAS-TDH3p-PgUGT71A53-FBA1t*)	[9]
Re-01	Rg1-02(*yorw17*:: *UAS-TDH3p-VvRHM-NRS-CYC1t,ble, yorw15*:: *TEF1p-synPgURT94m1-ENO2t,nat*)	This study

Supplementary Table 4. Primers used for the construction of yeast strains in this study

strains	Primers	Sequences (5’ to 3’)
Rg2-01	Rg201-01	TCGAGGAGAACTTCTAGTATATTC
	Rg201-02	CGCTTGACATCTACTATATGTAAGTATACGGCCCCATGAAGCAGGTGTTGTTGTCTG
	Rg201-03	GCTAACTCTCAACAGACAACAACACCTGCTTCATGGGGCCGTATACTTACATATAGT
	Rg201-04	TGTTCTTTGGCTTATACGTAGTATCATCCATGTTTAGTTAATTATAGTTCGTTGAC
	Rg201-05	TATACGGTCAACGAACTATAATTAACTAAACATGGATGATACTACGTATAAGCCAAAGA
	Rg201-06	ATCATTAAAGTAACTTAAGGAGTTAAATTTATCAGGTTCTCTTGTTTGGTTCAAAGAC
	Rg201-07	ATACGTCTTTGAACCAAACAAGAGAACCTGATAAATTTAACTCCTTAAGTTACT
	Rg201-08	AGGTTTTGGGACGCTCGAAGGCTTTAATTTGCGCGAAAAGCCAATTAGTGTGATAC
	Rg201-09	AGCACTTAGTATCACACTAATTGGCTTTTCGCGCAAATTAAAGCCTTCGAGCGTC
	Rg201-10	CATTGGCGAAGCTATTTGAGGAAATTATGTAAATCATGTAATTAGTTATGTCACGC
	Rg201-11	TGAATGTAAGCGTGACATAACTAATTACATGATTTACATAATTTCCTCAAATAGCT
	Rg201-12	GCATAGCAATCTAATCTAAGTTTTAATTACAAAATGAAGTCAGAATTGATATTCTTGC
	Rg201-13	GACGGGCAAGAATATCAATTCTGACTTCATTTTGTAATTAAAACTTAGATTAGA
	Rg201-14	AAGGAGTAGAAACATTTTGAAGCTATGGCGCGATAGCTTCAAAATGTTTCTACTCCT
	Rg201-15	GTAAAAAAGGAGTAGAAACATTTTGAAGCTATCGCGCCATAGCTTCAAAATGTTTCT
	Rg201-16	CTTGATCCTGGTCTTCTCGTTGGTGTCCATTTTGTTTGTTTATGTGTGTTTATTCG
	Rg201-17	TAGTTTCGAATAAACACACATAAACAAACAAAATGGACACCAACGAGAAGACCAGGA
	Rg201-18	TAAAAAACTATATCAATTAATTTGAATTAACTTTATGGACACCTCTTACCTTGACCTG
	Rg201-19	GCCGCAGGTCAAGGTAAGAGGTGTCCATAAAGTTAATTCAAATTAATTGATATAGT
	Rg201-20	GATTCGATACTAACGCCGCCATCCAGTGTCGAAGTAAGCTACTATGAAAGACTTTAC
	Rg201-21	GAGTTCTTTGTAAAGTCTTTCATAGTAGCTTACTTCGACACTGGATGGCGGCGTTAG

	Rg201-22	AGTAGATGATAGTTGATTTCTATTCCAACAAGCTTGCCTTGTCCCCGCCGGGTCA
	Rg201-23	CGGGTGACCCGGCGGGGACAAGGCAAGCTTGTTGGAATAGAAATCAACTATC
	Rg201-24	GGATATAGGAATCCTCAAAATG
Rg2-02,03	Rg202-04	TCAAGATGTTCTTTGGGGTGTGGGTAGCCATGTTTAGTTAATTATAGTTCGTTGAC
	Rg202-05	TATACGGTCAACGAACTATAATTAACTAAACATGGCTACCCACACCCCAAAGAACA
	Rg202-06	ATCATTAAAGTAACTTAAGGAGTTAAATTTATCAGGCCTTGACCTCGGTCTTCTTG
	Rg202-07	GCCAAACAAGAAGACCGAGGTCAAGGCCTGATAAATTTAACTCCTTAAGTTACT
Rg2-04	Rg204-01	GAACTGGGTTACCCGGGGCACCTGTC
	Rg204-02	GTCGATTCGATACTAACGCCGCCATCCAGTGTCGATTTCCTCTAATCAGGTTCCACCA
	Rg204-03	CGGGGTATCTGTTTGGTGGAACCTGATTAGAGGAAATCGACACTGGATGGCGGCGTTAG
	Rg204-04	AGGAGTAGAAACATTTTGAAGCTATGGCGCGAGCTTGCCTTGTCCCCGCCGGGTCAC
	Rg204-05	GCCGGGTGACCCGGCGGGGACAAGGCAAGCTCGCGCCATAGCTTCAAAATGTTTCTA
	Rg204-06	GACGGGCAAGAATATCAATTCTGACTTCATTTTGTTTGTTTATGTGTGTTTATTCG
	Rg204-07	TAGTTTCGAATAAACACACATAAACAAACAAAATGAAGTCAGAATTGATATTCTTGC
	Rg204-08	GAAAAGACTAATAATTCTTAGTTAAAAGCACTTTATTACATAATTTCCTCAAATAGC
	Rg204-09	TGGCGAAGCTATTTGAGGAAATTATGTAATAAAGTGCTTTTAACTAAGAATTAT
	Rg204-10	ACCTTTAGACTTACGTTTGCTACTCTCATAGGTATCATCTCCATCTCCCATATGC
	Rg204-11	TGCATATGGGAGATGGAGATGATACCTATGAGAGTAGCAAACGTAAGTCTAAAGGT
	Rg204-12	CTCACTATTTTTTACTGCGGAAGCGG
Re-01	Re01-01	GTCTACAGAATATACTAGATGTCCTC
	Re01-02	GAGTAGAAACATTTTGAAGCTATGGCGCGGGCATGAGTTATGGTTGCACAGTTACCA
	Re01-03	TAGATTTGGTAACTGTGCAACCATAACTCATGCCCGCGCCATAGCTTCAAAATGTTTC
	Re01-04	CAAGATGTTCTTTGGGGTGTGGGTAGCCATTTTGTTTGTTTATGTGTGTTTATTCG
	Re01-05	CTTAGTTTCGAATAAACACACATAAACAAACAAAATGGCTAC

		CCACACCCCAAAGAAC
	Re01-06	GCGTGAATGTAAGCGTGACATAACTAATTACATGATTCAGGCCTTGACCTCGGTCTTC
	Re01-07	CGAGCCAAACAAGAAGACCGAGGTCAAGGCCTGAATCATGTAATTAGTTATGTCACGC
	Re01-08	CGGGTGACCCGGCGGGGACGAGGCAAGCTTCTCAAGCAAGGTTTTCAGTATAATG
	Re01-09	GTAACATTATACTGAAAACCTTGCTTGAGAAGCTTGCCTCGTCCCCGCCGGGTCA
	Re01-10	CTTCCTATATGCATTTAAATGTGATGAATTTTGATCGACACTGGATGGCGGCGTTAGT
	Re01-11	GATACTAACGCCGCCATCCAGTGTCGATCAAAATTCATCACATTTAAATGCATATAGGA
	Re01-12	GCTGGCTCCCCTTAGACAAATACGC
	Re01-13	TCAATCAAAGCAACCCACAAATCCT
	Re01-14	GATATGCATATGGGAGATGGAGATGATACCTTGCGGTGTAAGAAAATGACATAAAGT
	Re01-15	CAAACTTTATGTCATTTTCTTACACCGCAAGGTATCATCTCCATCTCCCATATGCA
	Re01-16	GCCGCAGGTCAAGGTAAGAGGTGTCCATAATAAAGTGCTTTTAACTAAGAAT
	Re01-17	GACTAATAATTCTTAGTTAAAAGCACTTTATTATGGACACCTCTTACCTTGAC
	Re01-18	ATAGCAATCTAATCTAAGTTTTAATTACAAAATGGACACCAACGAGAAGACCAG
	Re01-19	ATCCTGGTCTTCTCGTTGGTGTCCATTTTGTAATTAAAACTTAGATTAGATTGC
	Re01-20	GCCGGGTGACCCGGCGGGGACAAGGCAAGCTATAGCTTCAAAATGTTTCTACTCCT
	Re01-21	AGTAAAAAAGGAGTAGAAACATTTTGAAGCTATAGCTTGCCTTGTCCCCGCCGGGT
	Re01-22	GATTTATAATGGTTTATCGGTTGCATTTTCCATGTCGACACTGGATGGCGGCGTTAGT
	Re01-23	ATTCGATACTAACGCCGCCATCCAGTGTCGACATGGAAAATGCAACCGATAAACCA
	Re01-24	GCCGTCCTCATGATGTGTTAGTTATAC

Supplementary Table 5. Genes used for the construction of yeast strains in this study

Genes	NCBI accession No. or RDBSB*NGDC accession No.
PgUGT71A54	*KP795113.1*
PgUGT71A53	*KF377585.1*
AtRHM2	*Q9LPG6.1*
synPgURT94	*OENC366046*
synPgURT94m1	*OENC366047*

***:** RDBSB, the Registry and Database of Bioparts for Synthetic Biology (https://www.biosino.org/rdbsb)

Supplementary Table 6. The yields of the triterpenoid products of yeast strains by shake flask and fed-batch fermentation.

	**Strains**	**PPD**	**DMG**	**CK**	**PPT**	**F1**	**Rg2**	**Rh1**	**Re**	**Rg1**
Shake flask (mg/L)	PPT-10	236.8 ± 3.5	-	-	401.8 ± 18.0	-	-	-	-	-
	Rg2-01	128.9 ± 4.7	-	-	274.5 ± 10.3	-	36.8 ± 1.3	-	-	-
	Rg2-02	222.0 ± 12.2	-	-	326.8 ± 12.3	-	66.4 ± 1.5	-	-	-
	Rg2-03	224.0 ± 38.1	-	-	336.4 ± 25.7	-	107.5 ± 3.9	-	-	-
	Rg2-04	151.8 ± 20.7	-	-	263.3 ± 33.2	-	147.1 ± 6.3	-	-	-
	Rg1-02	77.9 ± 4.7	109.3 ± 9.6	28.0 ± 1.0	97.8 ± 2.9	24.9 ± 1.1	-	-	-	126.9 ± 4.7
	Re-01	74.4 ± 2.9	69.6 ± 2.9	32.9 ±1.8	59.8 ± 4.4	10.6 ± 0.1	-	-	215.0 ± 12.0	2.8 ± 4.0
Fed-batch fermentation (g/L)	Rg2-04	2.2	-	-	3.0	-	1.3	-	-	-
	Re-01	1.0	0.5	0.7	0.2	0.1	-	-	3.6	0.2

“-” indicate not detected.

Supplementary Table 7. Enzyme conversion rate of synPgURT94 and synPgURT94m1 for the catalyze of Rh1 and Rg1 to yield Rg2 and Re. All data represent the mean of n = 3 biologically independent samples and errors show standard deviation.

	**Rg2 conversion %**	**Re conversion %**
synPgURT94	70.6 ± 2.6	95.5 ± 2.4
synPgURT94m1	92.4 ± 3.5	99.7 ± 3.0

Supplementary Table 8. Determination of enzyme kinetics of PgUGT71A53 and PgUGT71A54 toward PPT or F1 using UDP-glucose as the sugar donor.

All data represent the mean of n = 3 biologically independent samples and errors show standard deviation.

**Enzymes**	**Substrate and product**	***Vmax*** **(nmol/min/mg)**	***Km*** **(mM)**	***kcat*** **(s^{-1})**	***kcat/Km*** **(mM^{-1s-1})**	**References**
PgUGT71A54	PPT / Rh1	$1.99\times10^{3} \pm 4.33\times10^{2}$	$6.83\times10^{1} \pm 1.43\times10^{1}$	$1.76 \pm 3.80\times10^{-1}$	$2.58\times10^{-2} \pm 2.03\times10^{-4}$	[9]
PgUGT71A53	PPT / F1	$1.02\times10^{3} \pm 8.18\times10^{1}$	$6.88\times10^{-2} \pm 1.83\times10^{-2}$	$8.61\times10^{-1} \pm 6.92\times10^{-2}$	$1.11\times10^{1} \pm 4.00\times10^{-2}$	[9]
PgUGT71A54	F1 / Rg1	$1.59\times10^{1} \pm 8.64\times10^{-1}$	$1.76\times10^{-2} \pm 1.31\times10^{-3}$	$1.38\times10^{-2} \pm 7.52\times10^{-4}$	$7.87\times10^{-1} \pm 1.70\times10^{-2}$	this study
PgUGT71A53	F1 / Rg1	$2.13\times10^{1} \pm 2.25$	$2.62\times10^{-2}\pm7.29\times10^{-3}$	$1.89\times10^{-3} \pm 2.00\times10^{-4}$	$7.40\times10^{-2} \pm 1.30\times10^{-2}$	this study

Supplementary Table 9. Examples for the promotion of ginsenosides production via fed-batch fermentation in bioreactors.

Ginsenosides	Hosts	Shake flask fermentation (mg/L)	Fed-batch fermentation (g/L)	References
Rh2	*Saccharomyces cerevisiae*	179.3	2.3	[10]
CK	*S. cerevisiae*	261.6	5.7	[11]
Rg1	*S. cerevisiae*	105.0	2.0	[9]
NgR1	*S. cerevisiae*	114.0	1.6	[9]
NgR2	*S. cerevisiae*	42.0	1.3	[9]

Supplementary Table 10. *Panax ginseng* transcriptome dataset used for assembly.

#sample	sequence	qual	catalog
SRR2952867	SRR2952867_1.fastq,SRR2952867_2.fastq	33	*Panax_ginseng*
SRR2952868	SRR2952868_1.fastq,SRR2952868_2.fastq	33	*Panax_ginseng*
SRR2952869	SRR2952869_1.fastq,SRR2952869_2.fastq	33	*Panax_ginseng*
SRR2952870	SRR2952870_1.fastq,SRR2952870_2.fastq	33	*Panax_ginseng*
SRR2952871	SRR2952871_1.fastq,SRR2952871_2.fastq	33	*Panax_ginseng*
SRR2952872	SRR2952872_1.fastq,SRR2952872_2.fastq	33	*Panax_ginseng*
SRR2952873	SRR2952873_1.fastq,SRR2952873_2.fastq	33	*Panax_ginseng*
SRR2952874	SRR2952874_1.fastq,SRR2952874_2.fastq	33	*Panax_ginseng*
SRR2952875	SRR2952875_1.fastq,SRR2952875_2.fastq	33	*Panax_ginseng*
SRR2952876	SRR2952876_1.fastq,SRR2952876_2.fastq	33	*Panax_ginseng*
SRR2952877	SRR2952877_1.fastq,SRR2952877_2.fastq	33	*Panax_ginseng*
SRR2952878	SRR2952878_1.fastq,SRR2952878_2.fastq	33	*Panax_ginseng*
SRR2952879	SRR2952879_1.fastq,SRR2952879_2.fastq	33	*Panax_ginseng*
SRR2952880	SRR2952880_1.fastq,SRR2952880_2.fastq	33	*Panax_ginseng*
SRR2952881	SRR2952881_1.fastq,SRR2952881_2.fastq	33	*Panax_ginseng*
SRR2952882	SRR2952882_1.fastq,SRR2952882_2.fastq	33	*Panax_ginseng*
SRR2952883	SRR2952883_1.fastq,SRR2952883_2.fastq	33	*Panax_ginseng*
SRR2952884	SRR2952884_1.fastq,SRR2952884_2.fastq	33	*Panax_ginseng*
SRR5289466	SRR5289466_1.fastq,SRR5289466_2.fastq	33	*Panax_ginseng*
SRR5289467	SRR5289467_1.fastq,SRR5289467_2.fastq	33	*Panax_ginseng*
SRR5289468	SRR5289468_1.fastq,SRR5289468_2.fastq	33	*Panax_ginseng*
SRR5289469	SRR5289469_1.fastq,SRR5289469_2.fastq	33	*Panax_ginseng*
SRR5289470	SRR5289470_1.fastq,SRR5289470_2.fastq	33	*Panax_ginseng*
SRR5289471	SRR5289471_1.fastq,SRR5289471_2.fastq	33	*Panax_ginseng*
SRR5289472	SRR5289472_1.fastq,SRR5289472_2.fastq	33	*Panax_ginseng*
SRR5289473	SRR5289473_1.fastq,SRR5289473_2.fastq	33	*Panax_ginseng*
SRR5289474	SRR5289474_1.fastq,SRR5289474_2.fastq	33	*Panax_ginseng*

Supplementary Table 11. Ten *Panax ginseng* transcriptome datasets used for co-assembly to obtain the second transcripts dataset.

#sample	sequence	qual	catalog
SRR5289466	SRR5289466_1.fastq,SRR5289466_2.fastq	33	*Panax_ginseng*
SRR5289467	SRR5289467_1.fastq,SRR5289467_2.fastq	33	*Panax_ginseng*
SRR5289468	SRR5289468_1.fastq,SRR5289468_2.fastq	33	*Panax_ginseng*
SRR5289469	SRR5289469_1.fastq,SRR5289469_2.fastq	33	*Panax_ginseng*
SRR5289470	SRR5289470_1.fastq,SRR5289470_2.fastq	33	*Panax_ginseng*
SRR5289471	SRR5289471_1.fastq,SRR5289471_2.fastq	33	*Panax_ginseng*
SRR5289472	SRR5289472_1.fastq,SRR5289472_2.fastq	33	*Panax_ginseng*
SRR5289473	SRR5289473_1.fastq,SRR5289473_2.fastq	33	*Panax_ginseng*
SRR5289474	SRR5289474_1.fastq,SRR5289474_2.fastq	33	*Panax_ginseng*

Supplementary References

1. Yang, C., et al. The unprecedented diversity of UGT94-family UDP-glycosyltransferases in *Panax* plants and their contribution to ginsenoside biosynthesis. *Sci. Rep*. **10**(1): p. 15394（2020）.
2. Zhang, M., Liu, H., Wang, Q., Liu, S.&Zhang, Y. The 3-hydroxy-3-methylglutaryl-coenzyme A reductase 5 gene from *Malus domestica* enhances oxidative stress tolerance in *Arabidopsis thaliana*. *Plant Physiol. Biochem*. **146**: p. 269-277（2020）.
3. Leber, R., et al. A novel sequence element is involved in the transcriptional regulation of expression of the ERG1 (squalene epoxidase) gene in *Saccharomyces cerevisiae*. *Eur. J. Biochem*. **268**(4): p. 914-24（2001）.
4. Han, J.Y., Hwang, H.S., Choi, S.W., Kim, H.J.&Choi, Y.E. Cytochrome P450 CYP716A53v2 catalyzes the formation of protopanaxatriol from protopanaxadiol during ginsenoside biosynthesis in *Panax ginseng*. *Plant Cell Physiol*. **53**(9): p. 1535-45（2012）.
5. Han, J.Y., Kim, H.J., Kwon, Y.S.&Choi, Y.E. The Cyt P450 enzyme CYP716A47 catalyzes the formation of protopanaxadiol from dammarenediol-II during ginsenoside biosynthesis in *Panax ginseng*. *Plant Cell Physiol*. **52**(12): p. 2062-73（2011）.
6. Usadel, B., Kuschinsky, A.M., Rosso, M.G., Eckermann, N.&Pauly, M. RHM2 is involved in mucilage pectin synthesis and is required for the development of the seed coat in *Arabidopsis*. *Plant Physiol*. **134**(1): p. 286-95（2004）.
7. Pei, J., et al. Construction of a novel UDP-rhamnose regeneration system by a two-enzyme reaction system and application in glycosylation of flavonoid. *Biochem. Eng. J*. **139**: p. 33-42（2018）.
8. Kang, O.J.&Kim, J.S. Comparison of ginsenoside contents in different parts of korean ginseng (*Panax ginseng* C.A. Meyer). *Prev. Nutr. Food Sci*. **21**(4): p. 389-392（2016）.
9. Li, X., et al. High-level sustainable production of the characteristic protopanaxatriol-type saponins from Panax species in engineered *Saccharomyces cerevisiae*. *Metab. Eng*. **66**: p. 87-97（2021）.
10. Wang, P., et al. Synthesizing ginsenoside Rh2 in *Saccharomyces cerevisiae* cell factory at

high-efficiency. *Cell Discov*. **5**: p. 5（2019）.

11. Wang, P., Wang, J., Zhao, G., Yan, X.&Zhou, Z. Systematic optimization of the yeast cell factory for sustainable and high efficiency production of bioactive ginsenoside compound K. *Synth. Syst. Biotechnol*. **6**(2): p. 69-76（2021）.
