# Supplementary material for: Pathway elucidation of bioactive rhamnosylated ginsenosides in Panax ginseng and their de novo high-level production by engineered Saccharomyces cerevisiae
Source: Commun Biol. 2022 Aug 2;5:775. doi: 10.1038/s42003-022-03740-y (PMC9345943; doi:10.1038/s42003-022-03740-y)
Supplement: Supplementary file 3 — Reporting Summary [file 42003_2022_3740_MOESM3_ESM.pdf]

nature portfolio

Corresponding author(s): Pingping Wang

Last updated by author(s): Mar 8, 2022

Reporting Summary

Nature Portfolio wishes to improve the reproducibility of the work that we publish. This form provides structure for consistency and transparency in reporting. For further information on Nature Portfolio policies, see our Editorial Policies and the Editorial Policy Checklist.

Statistics

For all statistical analyses, confirm that the following items are present in the figure legend, table legend, main text, or Methods section.

n/a	Confirmed	
☐	☒	The exact sample size (n) for each experimental group/condition, given as a discrete number and unit of measurement
☐	☒	A statement on whether measurements were taken from distinct samples or whether the same sample was measured repeatedly
☒	☐	The statistical test(s) used AND whether they are one- or two-sided<br>*Only common tests should be described solely by name; describe more complex techniques in the Methods section.*
☒	☐	A description of all covariates tested
☒	☐	A description of any assumptions or corrections, such as tests of normality and adjustment for multiple comparisons
☐	☒	A full description of the statistical parameters including central tendency (e.g. means) or other basic estimates (e.g. regression coefficient) AND variation (e.g. standard deviation) or associated estimates of uncertainty (e.g. confidence intervals)
☒	☐	For null hypothesis testing, the test statistic (e.g. F, t, r) with confidence intervals, effect sizes, degrees of freedom and P value noted<br>*Give P values as exact values whenever suitable.*
☒	☐	For Bayesian analysis, information on the choice of priors and Markov chain Monte Carlo settings
☒	☐	For hierarchical and complex designs, identification of the appropriate level for tests and full reporting of outcomes
☒	☐	Estimates of effect sizes (e.g. Cohen's d, Pearson's r), indicating how they were calculated

Our web collection on statistics for biologists contains articles on many of the points above.

Software and code

Policy information about availability of computer code

Data collection	HPLC data was collected from a Shimadzu LC20A system equipped with a LC20ADXR pumper, an auto-sampler and a diode array detector; MS data was obtain by Agilent UHPLC-1290/MS QT0F6545A system equipped with Welch Boltimate C18 column (2.1*100 mm; 2.7µm); AVANCE III 400MHz Digital NMR Spectrometer was employed for 1H NMR data acquisition.
Data analysis	Trinity (version 2.5.1) for transcriptomic datasets assembly; Newbler (version3.0) for co-assembly; FASTQC (version 0.11.9) was used to assess the quality of the raw data, and Trimmomatic (version 0.39) was used to remove the connector and end low quality sequences.HPLC analysis was performed on a Shimadzu LC20A system.

For manuscripts utilizing custom algorithms or software that are central to the research but not yet described in published literature, software must be made available to editors and reviewers. We strongly encourage code deposition in a community repository (e.g. GitHub). See the Nature Portfolio guidelines for submitting code & software for further information.

Data

Policy information about availability of data

All manuscripts must include a data availability statement. This statement should provide the following information, where applicable:

- Accession codes, unique identifiers, or web links for publicly available datasets
- A description of any restrictions on data availability
- For clinical datasets or third party data, please ensure that the statement adheres to our policy

All raw sequencing data is deposited in the SRA, their accession number are listed in supplementary table 10 and 11. Gene sequences are deposited in NCBI or NGDC (https://www.biosino.org/npbiosys/), their accession number are listed in supplementary table 5. All data in figures has been made available in the supplementary source data file indicated in the Data Availability Statement.

Field-specific reporting

Please select the one below that is the best fit for your research. If you are not sure, read the appropriate sections before making your selection.

☒ Life sciences ☐ Behavioural & social sciences ☐ Ecological, evolutionary & environmental sciences

For a reference copy of the document with all sections, see nature.com/documents/nr-reporting-summary-flat.pdf

Life sciences study design

All studies must disclose on these points even when the disclosure is negative.

Sample size	Biological triplicates were chosen as the standard for the experiment.
Data exclusions	No data exclusion.
Replication	All determinations were repeated in biological triplicates, except for fed-batch fermentation.
Randomization	3 positive yest colonies were randomly selected for the analysis of ginsenoside production.
Blinding	N/A

Reporting for specific materials, systems and methods

We require information from authors about some types of materials, experimental systems and methods used in many studies. Here, indicate whether each material, system or method listed is relevant to your study. If you are not sure if a list item applies to your research, read the appropriate section before selecting a response.

Materials & experimental systems

n/a	Involved in the study	
☒	☐	Antibodies
☒	☐	Eukaryotic cell lines
☒	☐	Palaeontology and archaeology
☒	☐	Animals and other organisms
☒	☐	Human research participants
☒	☐	Clinical data
☒	☐	Dual use research of concern

Methods

n/a	Involved in the study	
☒	☐	ChIP-seq
☒	☐	Flow cytometry
☒	☐	MRI-based neuroimaging
